# Hypercoagulability Impairs Plaque Stability in Diabetes-Induced Atherosclerosis

**DOI:** 10.3390/nu14101991

**Published:** 2022-05-10

**Authors:** Saira Ambreen, Sameen Fatima, Ahmed Elwakiel, Rajiv Rana, Kunal Singh, Anubhuti Gupta, Dheerendra Gupta, Hamzah Khawaja, Jayakumar Manoharan, Christian Besler, Ulrich Laufs, Shrey Kohli, Berend Isermann, Khurrum Shahzad

**Affiliations:** 1 Institute of Laboratory Medicine, Clinical Chemistry and Molecular Diagnostic, University Hospital Leipzig, 04103 Leipzig, Germany; saira.ambreen@medizin.uni-leipzig.de (S.A.); sameen.fatima@medizin.uni-leipzig.de (S.F.); ahmed.elwakiel@medizin.uni-leipzig.de (A.E.); rajiv.rana@medizin.uni-leipzig.de (R.R.); kunal.singh@medizin.uni-leipzig.de (K.S.); anubhuti.gupta@medizin.uni-leipzig.de (A.G.); dheerendra.gupta@medizin.uni-leipzig.de (D.G.); hamzah.khawaja@medizin.uni-leipzig.de (H.K.); jayakumar.manoharan@medizin.uni-leipzig.de (J.M.); shrey.kohli@medizin.uni-leipzig.de (S.K.); berend.isermann@medizin.uni-leipzig.de (B.I.); 2 Institute of Experimental Internal Medicine, Medical Faculty, Otto von Guericke University, Leipziger Str. 44, 39120 Magdeburg, Germany; 3 Cardiology, Leipzig Heart Center, University of Leipzig, 04289 Leipzig, Germany; christian.besler@medizin.uni-leipzig.de; 4 Klinik und Poliklinik für Kardiologie, University Hospital Leipzig, 04103 Leipzig, Germany; ulrich.laufs@medizin.uni-leipzig.de

**Keywords:** atherosclerosis, diabetes, hypercoagulability, activated protein C, macrophages, smooth muscle cells

## Abstract

Diabetes mellitus, which is largely driven by nutritional and behavioral factors, is characterized by accelerated atherosclerosis with impaired plaque stability. Atherosclerosis and associated complications are the major cause of mortality in diabetic patients. Efficient therapeutic concepts for diabetes-associated atherosclerosis are lacking. Atherosclerosis among diabetic patients is associated with reduced endothelial thrombomodulin (TM) expression and impaired activated protein C (aPC) generation. Here, we demonstrate that atherosclerotic plaque stability is reduced in hyperglycemic mice expressing dysfunctional TM (TM^Pro/Pro^ mice), which have a pro-coagulant phenotype due to impaired thrombin inhibition and markedly reduced aPC generation. The vessel lumen and plaque size of atherosclerotic lesions in the truncus brachiocephalic were decreased in diabetic TM^Pro/Pro^ ApoE^-/-^ mice compared to diabetic ApoE^-/-^ mice. While lipid accumulation in lesions of diabetic TM^Pro/Pro^ ApoE^-/-^ mice was lower than that in diabetic ApoE^-/-^ mice, morphometric analyses revealed more prominent signs of instable plaques, such as a larger necrotic core area and decreased fibrous cap thickness in diabetic TM^Pro/Pro^ ApoE^-/-^ mice. Congruently, more macrophages and fewer smooth muscle cells were observed within lesions of diabetic TM^Pro/Pro^ ApoE^-/-^ mice. Thus, impaired TM function reduces plaque stability, a characteristic of hyperglycemia-associated plaques, thus suggesting the crucial role of impaired TM function in mediating diabetes-associated atherosclerosis.

## 1. Introduction

Atherosclerotic cardiovascular disease (ASCVD), including coronary artery disease and its associated complications, such as myocardial infarction, is a main cause of mortality and morbidity worldwide [1]. Lifestyle factors and nutrition are independently associated with susceptibility to ASCVD. Intake of specific nutrients affects the development and progression of atherosclerosis in ASCVD patients [2]. In individuals with an elevated genetic risk of ASCVD, healthy lifestyle and nutrition reduces the risk for ASCVD events by about 50%, compared to individuals with an unhealthy lifestyle and nutrition [3,4,5]. Lifestyle and nutrition likewise increase the risk of and the course of diabetes mellitus [6,7]. The risk of ASCVD, in turn, is markedly increased in both type 1 and type 2 diabetic patients. ASCVD events occur earlier in patients with diabetes than in non-diabetic patients [8,9], underscoring the importance of a better understanding of the mechanisms promoting diabetes-associated ASCVD. Diabetes-associated ASCVD is characterized by a larger necrotic core area, thin-cap atheroma and a more pronounced inflammatory cell infiltrate independent of other risk factors, thus indicating a pathophysiology that is at least partially disjunct from that in non-diabetic individuals [10,11]. The mechanisms underlying the different disease courses in diabetic and non-diabetic patients remain unknown. Hyperglycemia exacerbates atherosclerosis progression and hinders plaque regression [12], with increased expression of proinflammatory genes and reduced M2-associated gene expression in macrophages [12]. Intriguingly, despite intensive lipid lowering, the probability of atherosclerosis and myocardial infarction remains increased in diabetic patients, suggesting that diabetes-specific mechanisms contribute to atherosclerosis independent of elevated blood lipids [12,13]. Optimal glycemic control provides beneficial effects in preventing microvascular complications of diabetes, such as kidney disease and retinopathy. However, glucose lowering has only minor effects in preventing major cardiovascular events, especially in patients with established ASCVD [8].

Diabetes mellitus is considered a hypercoagulable state. Coagulation activation markers, such as prothrombin activation fragment 1 + 2 and thrombin-anti-thrombin complexes, are elevated in diabetes mellitus. Conversely, the levels of the anticoagulant protease activated protein C (aPC) decline, and are inversely linked with the severity of coronary artery atherosclerosis, diabetes mellitus and cardiac ischemic injury in human patients [14]. Similarly, endothelial expression of thrombomodulin (TM) and the endothelial cell protein C receptor is reduced in endothelial cells of atherosclerotic plaque in coronary arteries [15,16,17,18,19]. In general, the effect of hypercoagulability on ASCVD appears to be dose-dependent, as slightly increased levels of thrombin generation are associated with reduced cardiovascular events—an effect lost at higher thrombin concentrations [20,21]. Accordingly, reports on the effect of hypercoagulability on atherosclerosis in mice are partially controversial [18,22,23,24,25,26,27]. In normoglycemic mice, hypercoagulability has been shown to promote plaque stability in hyperlipidemic mice [18]. Although hypercoagulability is repeatedly observed in diabetes settings, the contribution of hypercoagulability to diabetes-induced atherosclerosis has not been studied to date.

## 2. Materials and Methods

### 2.1. Reagents

The following antibodies were used in the current study: rabbit anti-alpha smooth muscle actin (α-SMA) (Abcam, Berlin, Germany); and rat anti-MOMA-2, rabbit anti-mouse IgG HRP (Abcam, Germany). The following secondary antibodies for immunofluorescence were used: FITC goat anti-rabbit IgG; and FITC rabbit anti-goat IgG (Vector Laboratories, Burlingame, CA, USA).

Other reagents were as follows: vectashield mounting medium with DAPI (Vector Laboratories, USA); streptozotocin (Enzo Life Sciences, Lorrach, Germany); saffron, Oil Red-O (Sigma–Aldrich, Darmstadt, Germany); accu-check test strips, accu-check glucometer; albumin fraction V, hematoxylin Gill II, agarose (Carl ROTH, Karlsruhe, Germany); aqueous mounting medium (ZYTOMED, Berlin, Germany); TRIzol Reagent and PBS (Life Technologies, Schwerte, Germany); RevertAid™ H Minus First Strand cDNA Synthesis kit (Fermentas, Leon-Rot, Germany); rompun 2% (Bayer, Leverkusen, Germany); ketamine 10% (beta-pharm, Augsburg, Germany), mouse IL-6, IL-1β and TNF-α ELISA kits (R & D system, Minneapolis, MN, USA).

### 2.2. Mice

ApoE^-/-^ (002052) mice were obtained from the Jackson Laboratory (Bar Harbor, ME, USA). TM^Pro/Pro^ mice have been described previously [16,28]. In the current study, we used littermates backcrossed for at least 10 generations on a C57BL/6J background. Only age-matched mice were used throughout the study. All animal experiments were conducted following standards and procedures approved by the local Animal Care and Use Committee (IKCP/G/05-1083/11_Maus, 30.01.2010, Landesverwaltungsamt Halle, Germany).

### 2.3. Atherogenic Mouse Models

Female ApoE^-/-^ or TM^Pro/Pro^ ApoE^-/-^ mice (age 6 to 8 weeks) were fed a normal chow diet and were made diabetic (DM) by injecting streptozotocin (STZ, 60 mg/kg, intraperitoneally, once daily for five consecutive days, freshly dissolved in 0.05 M sterile sodium citrate, pH 4.5), reflecting type 1 DM [29,30,31,32]. Control mice were injected with an equal volume of 0.05 M sodium citrate, pH 4.5, for 5 days. All mice were maintained on a normal chow diet [16,29,33]. Blood glucose and body weight were measured once weekly [16,29,33]. On average, 85–90% of mice became diabetic (blood glucose > 300 mg/dL) within the first 4 weeks, and these were included as diabetic mice in the experiments. Mice not developing persistently elevated blood glucose levels and maintaining blood glucose levels of <200 mg/dL despite STZ injections were included in the control group [16]. The endpoints analyzed did not differ between STZ-injected but normoglycemic mice and sodium-citrated injected mice. Hyperglycemia (minimum 300 mg/dL) was maintained for up to 22 weeks.

Atherosclerotic plaque morphology was analyzed using Image-Pro Plus software from Media Cybernetics as previously described [18]. Plaque characteristics were determined as follows:

- Aortic plaque score (0–4 Arbitrary units): Aortic plaque score was determined as described previously [34]. 0 = no lesions; 1 = Lesions only in bifurcation; 2 = like 1 + at least one long-stretch lesion; 3 = like 1 + at least two long-stretch lesions; 4 = like 1 + three to four long-stretch lesions.
- Vessel lumen (in μm^2^): the vessel lumen is the area within the blood vessel, consisting of both the remaining open lumen and the plaque area. It does not include the vessel wall itself.
- Total plaque size (in μm^2^): the size of the plaque comprising all parts of the atheroscleroma (fibrous cap, necrotic tissue, fibrous tissue, etc.) within the vessel lumen.
- Stenosis (in percent): the stenosis is determined as the relative proportion of the plaque size in relation to the total vessel lumen.
- Necrotic core area (in percent): the area stained blue upon MOVATs stain; given as the percentage of the total plaque size.
- Fibrous cap thickness (in μm): the fibrous cap thickness is the minimal thickness of the fibrous tissue overlaying a necrotic core. If multiple necrotic cores were present within one plaque, the thickness of all fibrous caps was determined, and the average was used for further analyses.

### 2.4. Analysis of Mice

After 22 weeks of age, the body weight of the mice was measured, and the mice were sacrificed [18,29,35]. Blood samples were obtained from the inferior vena cava of anticoagulated mice (500 U of unfractionated heparin, intraperitoneally). Blood was centrifuged at 2000× *g* for 20 min at 4 °C, and plasma was snap frozen in liquid nitrogen. Mice were perfused with ice-cold PBS for 10 min, and the heart and aortic arches, including brachiocephalic arteries, were embedded in Tissue-Tek^®^ O.C.T.™ compound and snap frozen. Brachiocephalic arteries were sectioned from distal to proximal at 5 μm thickness. The thoracic aorta was fixed in 4% buffered formalin for 20 min, washed twice in PBS for 10 min and stored for no more than one day at 4 °C before analysis.

### 2.5. Analysis of Blood Lipids

Plasma samples were analyzed in the accredited central laboratory of University Hospital Leipzig using standard operating procedures according to the manufacturers’ instructions. Cholesterol, high density lipoprotein and triglycerides were analyzed on a Siemens ADVIA Chemistry XPT System.

### 2.6. Histology and Immunohistochemistry

Oil Red O staining was conducted on thoracic aortae (opened longitudinally) or frozen sections of the brachiocephalic arteries [18,29,35]. Tissue was stained with Oil Red O for 10 min, and rinsed twice with distilled water for 20 s, and once in running tap water for 10 min. Aortae were pinned on a black wax surface using 0.1-mm-diameter stainless steel pins, as described previously [18,29,35]. Cryopreserved sections of the brachiocephalic arteries and aortic roots (4 μm) were fixed in ice-cold acetone for 2 min, rinsed twice in ice-cold 1 × PBS, and stained with Oil Red O using the same protocol as described above [18,29,35]. Frozen sections were counterstained with hematoxylin for 40 s, rinsed in tap water, and mounted with aqueous mount. Lipid-rich areas of the aortae were analyzed by a blinded investigator using Image-Pro Plus software, as described previously [18,29,35]. MOVAT staining was performed on frozen sections of brachiocephalic arteries. Frozen sections (4 µm) were fixed in Bouin’s solution at 50 °C for 10 min and stained with 5% sodium thiosulfate for 5 min, 1% Alcian blue for 15 min, alkaline alcohol for 10 min, Movat’s Weigerts solution for 20 min, crocein scarlet acid/fuchsin solution for 1 min, 5% phosphotungstic acid for 5 min and 1% acetic acid for 5 min. Between every staining step, the tissue sections were washed with tap water and distilled water. Afterward, they were dehydrated in 95% and 100% ethanol for 1 min and stained in alcohol saffron for 8 min. Brachiocephalic arteries were washed in 100% ethanol for 1 min, moved to xylol for 10 min and covered with a cytoseal mounting medium. Every 15th section (~90 μm) of the brachiocephalic arteries and aortic roots was analyzed to quantify the plaque area. For histological analysis, images were captured with an Olympus Bx43 microscope (Olympus, Hamburg, Germany). Image-Pro Plus software (version 6.0) software was used for image analysis [18,29,35].

For immunofluorescence, frozen sections of brachiocephalic arteries or aortic valves with maximum plaque size were fixed in ice-cold acetone for 8 min, washed twice with ice-cold PBS and incubated in 2% BSA in PBST for 1 h. Sections were then incubated overnight at 4 °C with primary antibodies against MOMA-2, or α-SMC actin. Sections incubated without primary antibodies were used as negative controls for background correction. After overnight incubation, the sections were washed three times with 1 × PBS for five minutes each, followed by incubation with fluorescently labeled corresponding secondary antibodies. After washing, nuclear counterstaining was conducted using mounting medium with DAPI. Images were visualized, captured and analyzed using a fluorescence microscope. All histological analyses were performed by two independent blinded investigators. Immunohistochemistry and immunofluorescence images were captured with an Olympus Bx43 microscope (Olympus, Hamburg, Germany). The relative macrophage and SMC contents within the lesions were quantified by measuring the immunostained-positive area using computer-assisted image analysis software (Image-Pro Plus; Media Cybernetics, Bethesda, MD, USA).

### 2.7. Reverse Transcriptase Polymerase Chain Reaction (RT–PCR)

RNA was extracted from aortic tissues (comprising the plaque and surrounding tissue) using an RNeasy mini kit (QIAGEN, Hilden, Germany). The RNA pellet was air-dried for 5 min and redissolved in 20 μL DEPC-water at 55 °C for 10 min. The RNA concentration was measured in a nanodrop (2000C, Peq lab, Erlangen, Germany), and a 1.8% agarose gel was run to verify the purity and integrity of the RNA. cDNA was synthesized according to the manufacturer’s protocol (SuperScript First-Strand Synthesis System for RT–PCR, Fermentas, Germany). Primers were custom synthesized by Thermo Fisher Scientific, and PCR was performed using Taq polymerase. Reactions lacking reverse transcriptase served as negative controls, and GAPDH was used as a housekeeping gene.

### 2.8. IL-1β, IL-6 and TNF-α Immunoassay

Mouse blood samples were obtained from the inferior vena cava in 0.38% sodium citrate. Plasma was obtained by centrifugation of blood samples for 10 min at 2000× *g* and RT. Plasma samples were stored at −80 °C until analyses. We measured the concentrations of mouse IL-1β, IL-6 and TNF-α (R&D Systems) by ELISA according to the manufacturer’s instructions [30,36,37].

### 2.9. Statistical Analysis

Female mice were grouped according to genotype, and randomly assigned to different interventions (control, streptozotocin). The data are summarized as the mean ± standard error of the mean (SEM). Statistical analyses were performed with the Mann–Whitney U test or analysis of variance (ANOVA), as appropriate. Post-hoc comparisons of ANOVA were corrected with the Bonferroni method, as indicated in the figure legend. The Kolmogorov–Smirnov test or D’Agostino-Pearson normality test was used to determine whether the data were consistent with a Gaussian distribution. Prism 8 (www.graphpad.com, accessed on 2 April 2020) software was used for statistical analyses. Values of *p* ≤ 0.05 were considered statistically significant.

## 3. Results

### 3.1. Comparable Plasma Lipid Levels in Hyperglycemic ApoE^-/-^ and TM^Pro/Pro^ ApoE^-/-^ Mice

**Figure 1:**
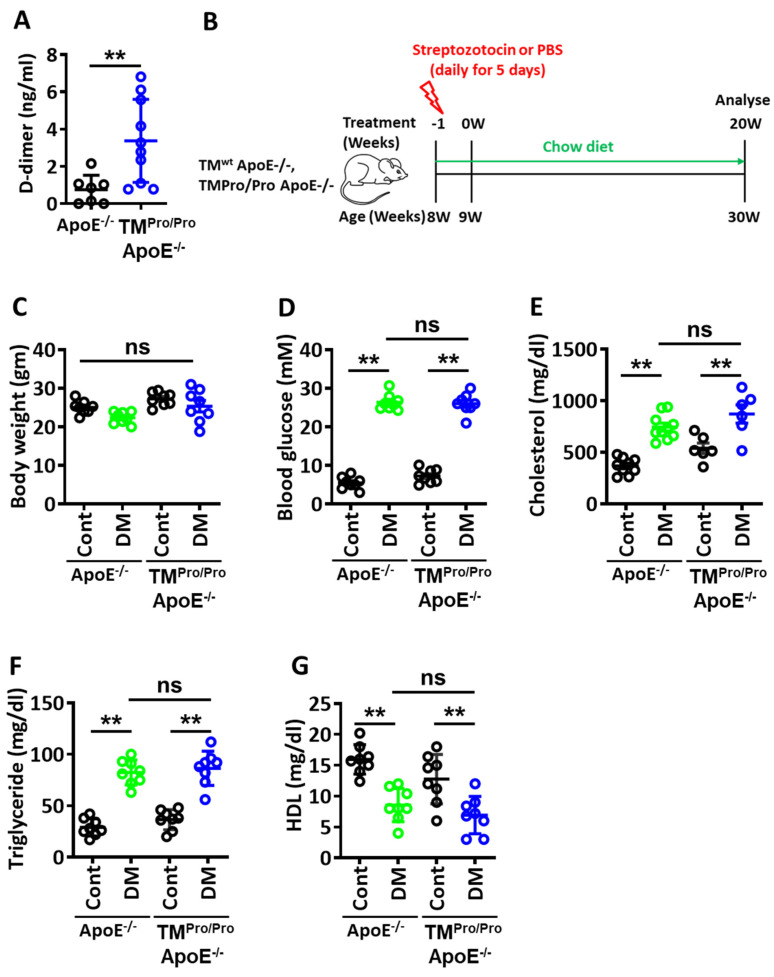
**Plasma D-dimer, body weight, blood glucose and plasma lipid levels**. (**A**) Plasma D-dimer levels. (**B**) Experimental design. Body weight (**C**), blood glucose levels (**D**), total plasma cholesterol levels (**E**), plasma triglyceride levels (**F**) and plasma HDL levels (**G**). ApoE^-/-^ and TM^Pro/Pro^ ApoE^-/-^ control mice (Cont, normal chow diet, citrate instead of streptozotocin injections), ApoE^-/-^ DM mice and TM^Pro/Pro^ ApoE^-/-^ DM (normal chow diet, streptozotocin injections). Each dot represents data obtained from one mouse specimen (n = at least 6 mice per group); ** *p* < 0.01; ns: non-significant; Mann–Whitney U test (**A**); two-way ANOVA (**C**–**G**).

To determine the effect of hypercoagulability on hyperglycemia-associated atherosclerotic plaque development, we directly compared diabetic ApoE^-/-^ mice expressing wild-type thrombomodulin (TM, *Thbd*) with diabetic ApoE^-/-^ mice expressing the TM^Pro^ mutant (TM^Pro/Pro^ mice). The Glu404Pro mutation mimics oxidative damage of the short domain between thrombomodulin’s EGF domains 4 and 5 and the associated marked reduction of thrombin inhibition and protein C activation. This mutation mimics loss of TM-function as observed in endothelial dysfunction and the associated hypercoagulability, reflected by an increased plasma TAT [18] and D-dimer levels (Figure 1A) and low activated protein C (aPC) plasma levels [28,38]. Mice were made hyperglycemic and followed up for 22 weeks on a chow diet (Figure 1B, see experimental scheme). Body weight was not different, while blood glucose levels differed between diabetic and non-diabetic ApoE^-/-^mice, as expected (Figure 1C,D). Plasma lipids were increased (total cholesterol, triglycerides) or decreased (HDL) in diabetic mice, but were comparable between diabetic ApoE^-/-^ and TM^Pro/Pro^ ApoE^-/-^ mice (Figure 1E–G).

### 3.2. Smaller but Unstable Plaques in Hyperglycemic aPC-Deficient ApoE^-/-^ Mice

**Figure 2:**
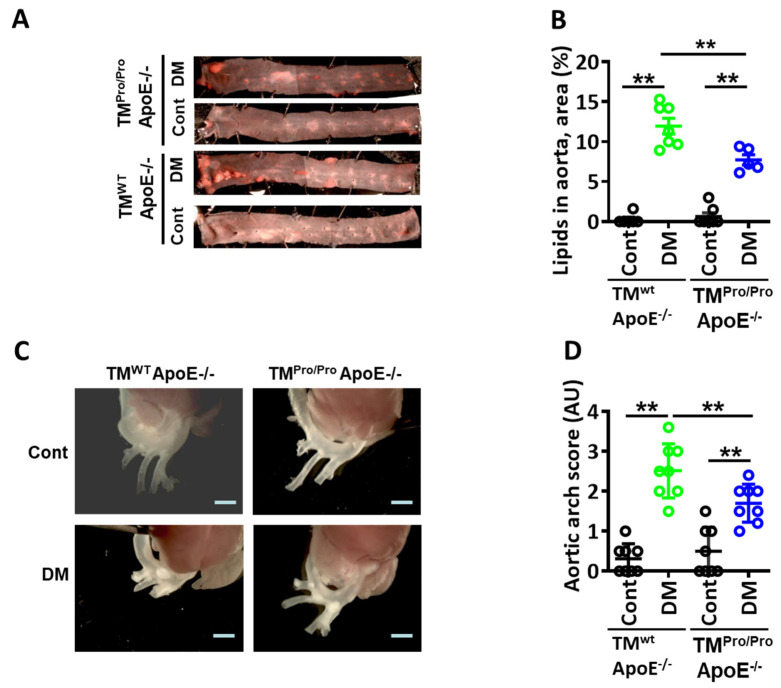
**Smaller plaques in hyperglycemic TM^Pro/Pro^ ApoE^-/-^ versus hyperglycemic ApoE^-/-^ mice.** (**A**,**B**): Representative images of thoracic aortae showing *en face* plaques as detected by Oil Red O staining (**A**) and dot-plot summarizing data (total area stained with Oil Red O, (**B**) in control (normal chow diet, Cont) and diabetic (STZ-induced hyperglycemia, DM) mice. Representative images showing plaques within the aortic arch vessel (**C**) and dot plot summarizing plaque score (**D**). ApoE^-/-^ and TM^Pro/Pro^ ApoE^-/-^ control mice (Cont, normal chow diet, citrate instead of streptozotocin injections), ApoE-/- DM mice and TM^Pro/Pro^ ApoE^-/-^ DM (normal chow diet, streptozotocin injections). Each dot represents data obtained from one mouse specimen (n = at least 6 mice per group); ** *p* < 0.01; two-way ANOVA.

**Figure 3:**
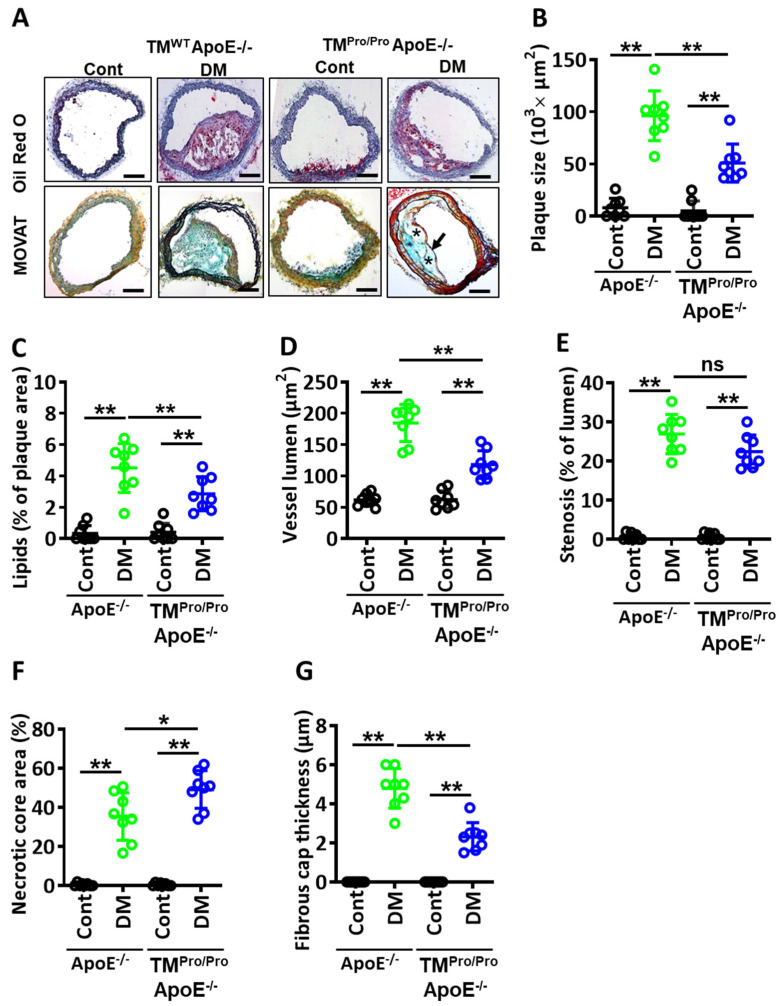
**Less stable plaques in hyperglycemic TM^Pro/Pro^ ApoE^-/-^ versus hyperglycemic ApoE^-/-^ mice.** (**A**,**B**) Representative images showing MOVATs staining (**A**, lower panel) and Oil Red O staining of brachiocephalic arteries (**A**, upper panel). Dot plot summarizing the plaque area data (**B**). Necrotic core area within plaque is indicated by a * and fibrous caps thickness by a black arrow. (**C**) Dot-plot summarizing lipid deposition within brachiocephalic arteries, Oil Red O-staining. (**D**,**E**) Dot plot summarizing the total vessel lumen area (**D**) and extent of stenosis (**E**) derived from morphometric analyses of MOVAT-stained images of brachiocephalic arteries. (**F**,**G**) Morphometric analyses of MOVATs stained images of brachiocephalic arteries reveal increased necrotic core area (**F**) and thinner fibrous caps (**G**), in TM^Pro/Pro^ ApoE^-/-^ DM mice compared to ApoE^-/-^ DM. The dot plots in b-f summarize the results obtained from MOVAT-stained brachiocephalic arteries. ApoE^-/-^ and TM^Pro/Pro^ ApoE^-/-^ control mice (Cont, normal chow diet, citrate instead of streptozotocin injections), ApoE-/- DM mice and TM^Pro/Pro^ ApoE^-/-^ DM (normal chow diet, streptozotocin injections). Each dot represents data obtained from one mouse specimen (n = 8, each group); ** *p* < 0.01, * *p* < 0.05; ns: non-significant; two-way ANOVA.

Analyses of Oil Red O-stained aortae *en face* revealed a significant decrease in lipid deposits in TM^Pro/Pro^ ApoE^-/-^ DM mice compared to ApoE^-/-^ DM mice (Figure 2A,B). Congruently, fewer plaques were observed within the aortic arch of TM^Pro/Pro^ ApoE^-/-^ DM mice than in ApoE^-/-^ DM mice (Figure 2C,D). Likewise, plaques in the brachiocephalic artery were smaller, and lipid content within plaques of the brachiocephalic artery was decreased in TM^Pro/Pro^ ApoE^-/-^ DM mice compared to ApoE^-/-^ DM mice (Figure 3A–C). Despite different plaque sizes, the total vessel lumen area **(**Figure 3D) and degree of stenosis (Figure 3E) did not differ between TM^Pro/Pro^ ApoE^-/-^ DM and ApoE^-/-^ DM mice.

In addition to plaque size and vascular stenosis, plaque stability is an important determinant of clinical outcome. We therefore evaluated plaque stability in ApoE^-/-^ DM and TM^Pro/Pro^ ApoE^-/-^ DM mice. Signs of plaque instability were more evident in TM^Pro/Pro^ ApoE^-/-^ DM mice than in ApoE^-/-^ DM mice. Thus, plaques in TM^Pro/Pro^ ApoE^-/-^ DM mice displayed signs of decreased plaque stability, such as an increased necrotic core area (Figure 3F) and thinner fibrous caps (Figure 3G).

### 3.3. More Macrophages and Fewer Smooth Muscle Cells within Plaques of Hyperglycemic TM^Pro/Pro^ ApoE^-/-^ Mice

Plaque morphology and stability depend at least in part on the cellular components present within the plaque and the associated inflammatory response. Hence, we next evaluated the cellular composition and parameters reflecting inflammation.

**Figure 4:**
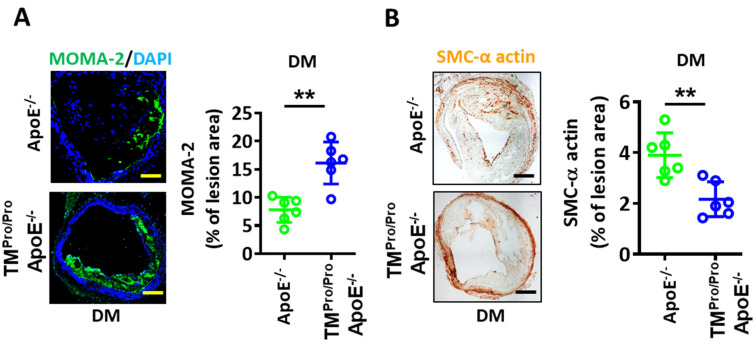
**More macrophages and proinflammatory cytokines andfewer smooth muscle cells in hyperglycemic TM^Pro/Pro^ ApoE^-/-^ versus hyperglycemic ApoE^-/-^ mice.** (**A**): Representative images showing immunofluorescence staining of macrophages (left panel, MOMA-2, green; DAPI nuclear counterstain, blue) and dot-plot summarizing data (right panel). (**B**): Representative immunohistochemically stained images showing smooth muscle cells (left panel, α-actin, positive cells detected by HRP-DAB reaction, brown) within lesions and dot-plot summarizing data (right panel). Each dot represents data obtained from one mouse specimen (n = 6, each group); ** *p* < 0.01, Student’s *t*-Test.

In plaques of TM^Pro/Pro^ ApoE^-/-^ DM mice, the macrophage area (immunohistochemically positive for MOMA-2) was increased (Figure 4A), while the area staining positive for smooth muscle cells (SMC α-actin positive area) was reduced in comparison to plaques of ApoE^-/-^ DM mice (Figure 4B). The observed shift to more macrophages and fewer SMCs corroborates the reduced plaque stability in TM^Pro/Pro^ ApoE^-/-^ DM mice.

**Figure 5:**
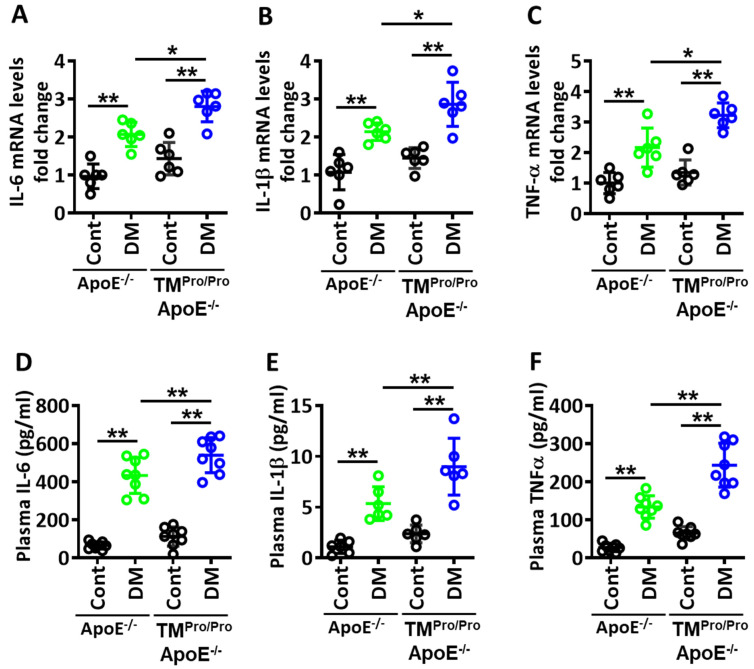
**More proinflammatory cytokines in hyperglycemic TM^Pro/Pro^ ApoE^-/-^ versus hyperglycemic ApoE^-/-^ mice**. (**A**–**C**): Dot plot summarizing data for real-time PCR showing aortic expression of IL-6 (**C**), IL-1β (**D**) and TNF-α (**E**). GAPDH was used as a housekeeping gene. (**D**–**F**): Dot plot summarizing data for plasma levels of IL-6 (**D**), IL-1β (**E**) and TNF-α (**F**). ApoE^-/-^ and TM^Pro/Pro^ ApoE^-/-^ control mice (Cont, normal chow diet, citrate instead of streptozotocin injections), ApoE^-/-^ DM mice and TM^Pro/Pro^ ApoE^-/-^ DM (normal chow diet, streptozotocin injections). Each dot represents data obtained from one mouse specimen (n = at least 6 mice per group); ** *p* < 0.01, * *p* < 0.05; ns: non-significant; two-way ANOVA.

Plaque-associated macrophages impair plaque stability in part by generating proinflammatory cytokines. Therefore, we next determined aortic mRNA expression (qPCR) of the proinflammatory cytokines interleukin 6 (IL-6), IL-1β and tumor necrosis factor alpha (TNF-α). Aortic mRNA expression levels of all these proinflammatory cytokines were markedly increased in TM^Pro/Pro^ ApoE^-/-^ DM mice compared to ApoE^-/-^ DM mice (Figure 5A–C). We then determined the plasma levels of these cytokines. In parallel, plasma levels of IL-6, IL-1β and TNF-α were induced in TM^Pro/Pro^ ApoE^-/-^ DM mice compared to ApoE^-/-^ DM mice, corroborating the mRNA data (Figure 5D–F).

In summary, impairment of TM function in TM^Pro/Pro^ ApoE^-/-^ DM mice results in smaller but unstable plaques, which are characterized by more macrophages, fewer SMCs and increased aortic expression and plasma levels of proinflammatory cytokines when compared to ApoE^-/-^ diabetic mice.

## 4. Discussion

Diabetes mellitus is closely associated with a hypercoagulable state. The majority of diabetic patients die due to cardiovascular complications, such as myocardial infarction, cerebrovascular events and peripheral vascular complications [39,40]. Reduced vascular thrombomodulin expression and aPC plasma levels have been associated with diabetes and associated vascular complications [15,16,17,18,19]. While impaired thrombomodulin-dependent protein C activation has been mechanistically linked with diabetic microangiopathy (e.g., nephropathy) [16,32,33,41,42], the role of impaired aPC activation and hypercoagulability in diabetic-associated macroangiopathy (i.e., atherosclerosis) remained unknown. Here, we demonstrate that partial loss of TM function, resulting in hypercoagulability and reduced aPC generation, promotes atherosclerotic plaque instability in hyperglycemic mice, as reflected by a larger necrotic core area, thin fibrous cap, fewer SMCs, more macrophages and increased proinflammatory cytokines. This reflects a plaque morphology associated with an increased risk for acute vascular complications, as observed frequently in diabetic patients [10,11]. As loss of endothelial TM function reflects endothelial dysfunction in diabetic patients, the current data suggest that impaired endothelial TM function drives accelerated atherosclerosis in diabetic patients.

Plasma levels of blood lipids were comparable in hyperglycemic ApoE^-/-^ and TM^Pro/Pro^ ApoE^-/-^ mice, raising the question of how loss of TM function accelerates macroangiopathy in hyperglycemic mice. Loss of TM function promotes thrombin generation, which conveys procoagulant and proinflammatory effects while suppressing aPC formation, an anticoagulant and anti-inflammatory protease. Congruently, we observed increased expression and plasma levels of proinflammatory cytokines. Hyperglycemia is known to promote unstable plaques through chronic inflammation [43,44]. The interaction of hyperglycemia and hypercoagulability may be bidirectional: a loss of endothelial TM function and associated hypercoagulability increases inflammation, while inflammatory cytokines contribute to hypercoagulation and abnormal clot formation in type 2 diabetes mellitus [45]. Accordingly, cytokines were higher in hypercoagulable diabetic mice than in non-hypercoagulable diabetic mice.

Inflammation has a critical function at all stages of the atherothrombotic process, during which immune mechanisms interact with metabolic risk factors to initiate and propagate atherosclerotic lesions [46]. Activated immune cells—neutrophils, macrophages, and T cells—within the plaque generate proinflammatory cytokines [47]. Additionally, NLRP3 inflammasome-associated activation of IL-1β and IL-18 has been identified as a key pathomechanism in ASCVD. Recently, the CANTOS trial showed that targeting IL-1β reduced ASCVD event rates in patients with diabetes without lowering lipids or blood pressure [48]. Importantly, the efficacy of IL-1β antibody was directly associated with the reduction in IL-6 and CRP, clinical biomarkers of inflammation [48]. Intriguingly, a very recent ongoing clinical trial targeting IL-6 has shown great promise for reducing cardiovascular events in high-risk patients [49]. Whether IL-6 conveys also convey anti-inflammatory effects, e.g., in low-risk cardiac patients, the importance of sIL-6R in mediating these effects needs to be evaluated [50].

The observed induction of IL-6, IL-1β and TNFα in hypercoagulable diabetic mice is consistent with these observations and with the perception of diabetic vascular complications as inflammation-driven diseases. Intriguingly, inhibition of proinflammatory signaling by aPC has previously been demonstrated in both atherosclerotic lesions of diabetic mice and infarcted heart tissue [29,36]. As aPC inhibits NF-κB (nuclear factor kappa-light-chain-enhancer of activated B cells) signaling [37,51,52] and as NF-κB induces the pro-inflammatory cytokines IL-6 and TNFα [53], we propose that aPC restricts NF-κB signaling to avert the production of inflammatory cytokines within atherosclerotic lesions in the setting of diabetes mellitus. Based on these data, we propose that the aggravated loss of endothelial function with loss of TM and reduced aPC generation in patients with diabetes mellitus contributes to accelerated atherosclerosis. aPC-based approaches may allow modulation of the proinflammatory phenotype in the context of diabetes-associated atherosclerosis.

Previous reports have shown partially opposing effects of hypercoagulability in TM^Pro/Pro^ mice on atherosclerosis in hyperlipidemic mice [18,22]. Although both studies showed aggravated atherosclerosis, opposing effects of hypercoagulability on plaque stability were reported [18,22]. These differences may be partially explained by the specific mouse models (high-fat diet versus high-fat diet plus surgical implantation of perivascular carotid collars) or the experimental time lines used [18,22]. Other potential explanations are differences in food compositions or microbiota secondary to the specific animal husbandry conditions [54,55]. Several studies have demonstrated a relationship between microbiota and atherosclerosis [54,55]. Our current data show that changes in the metabolic state (hyperlipidemia versus hyperglycemia) have an impact on plaque stability, supporting that additional factors impact the role of hypercoagulability in atherosclerosis. The delicate role of hypercoagulability in ASCVD is reflected by the dose-dependent (hormetic) effect of thrombin on cardiovascular outcome [20,21]. It is possible that hypercoagulability, as observed in diabetes mellitus, reverses the “hormetic” or low-dose protective effect of thrombin in regard to ASCVD, resulting in plaque instability [56,57].

While providing novel insights, the current study has potential limitations. Thus, due to restriction in the permission for in-vivo mouse work, we were only able to use female mice in the current study. Furthermore, in the current study, we used a mouse model of T1DM. Although the ApoE^-/-^ streptozotocin model is an established mouse model and considered to be a suitable model to study diabetes-associated atherosclerosis, we cannot exclude the possibility that metabolic changes typically observed in type 2 diabetic patients, such as obesity, increased insulin levels, or impaired insulin signaling, further affect plaque stability.

Despite these potential limitations, the current study demonstrates that endothelial dysfunction with loss of TM function and associated hypercoagulability and reduced aPC generation contributes to accelerated atherosclerosis in diabetes mellitus. Therapies restoring TM function, such as soluble TM [58,59,60,61,62] or mimicking the function of aPC, such as parmodulin or aPC-mimetics [36,63,64], may be suitable to specifically combat accelerated atherosclerosis in diabetes mellitus.

## Abbreviations

aPC	activated protein C
ApoE	apolipoprotein E
ASCVD	atherosclerotic cardiovascular disease
ANOVA	analysis of variance
DM	diabetes mellitus
DAPI	4′,6-diamidino-2-phenylindole
DEPC	diethyl pyrocarbonate
EPCR	endothelial protein C receptor
NF-κB	nuclear factor kappa-light-chain-enhancer of activated B cells
qPCR	quantitative polymerase chain reaction
ROS	reactive oxygen species
RT-PCR	reverse transcriptase polymerase chain reaction
SMC	smooth muscle cells
α-SMC	alpha-smooth muscle actin
SEM	standard error mean
STZ	streptozotocin
TM	thrombomodulin

## References

[1] Benjamin E.J., Muntner P., Alonso A., Bittencourt M.S., Callaway C.W., Carson A.P., Chamberlain A.M., Chang A.R., Cheng S., Das S.R. (2019). Heart Disease and Stroke Statistics-2019 Update: A Report From the American Heart Association. Circulation.

[2] Casas R., Estruch R., Sacanella E. (2018). Influence of Bioactive Nutrients on the Atherosclerotic Process: A Review. Nutrients.

[3] Khera A.V., Emdin C.A., Drake I., Natarajan P., Bick A.G., Cook N.R., Chasman D.I., Baber U., Mehran R., Rader D.J. (2016). Genetic Risk, Adherence to a Healthy Lifestyle, and Coronary Disease. N. Engl. J. Med..

[4] Abraham G., Havulinna A.S., Bhalala O.G., Byars S.G., De Livera A.M., Yetukuri L., Tikkanen E., Perola M., Schunkert H., Sijbrands E.J. (2016). Genomic prediction of coronary heart disease. Eur. Heart J..

[5] Said M.A., Verweij N., van der Harst P. (2018). Associations of Combined Genetic and Lifestyle Risks With Incident Cardiovascular Disease and Diabetes in the UK Biobank Study. JAMA Cardiol..

[6] Ardisson Korat A.V., Willett W.C., Hu F.B. (2014). Diet, lifestyle, and genetic risk factors for type 2 diabetes: A review from the Nurses’ Health Study, Nurses’ Health Study 2, and Health Professionals’ Follow-up Study. Curr. Nutr. Rep..

[7] Guo Y., Huang Z., Sang D., Gao Q., Li Q. (2020). The Role of Nutrition in the Prevention and Intervention of Type 2 Diabetes. Front. Bioeng. Biotechnol..

[8] Stitziel N.O., Kanter J.E., Bornfeldt K.E. (2020). Emerging Targets for Cardiovascular Disease Prevention in Diabetes. Trends Mol. Med..

[9] Maahs D.M., Daniels S.R., de Ferranti S.D., Dichek H.L., Flynn J., Goldstein B.I., Kelly A.S., Nadeau K.J., Martyn-Nemeth P., Osganian S.K. (2014). Cardiovascular disease risk factors in youth with diabetes mellitus: A scientific statement from the American Heart Association. Circulation.

[10] Sugiyama T., Yamamoto E., Bryniarski K., Xing L., Fracassi F., Lee H., Jang I.K. (2018). Coronary Plaque Characteristics in Patients With Diabetes Mellitus Who Presented With Acute Coronary Syndromes. J. Am. Heart Assoc..

[11] Yahagi K., Kolodgie F.D., Lutter C., Mori H., Romero M.E., Finn A.V., Virmani R. (2017). Pathology of Human Coronary and Carotid Artery Atherosclerosis and Vascular Calcification in Diabetes Mellitus. Arterioscler. Thromb. Vasc. Biol..

[12] Parathath S., Grauer L., Huang L.S., Sanson M., Distel E., Goldberg I.J., Fisher E.A. (2011). Diabetes adversely affects macrophages during atherosclerotic plaque regression in mice. Diabetes.

[13] Zeadin M.G., Petlura C.I., Werstuck G.H. (2013). Molecular mechanisms linking diabetes to the accelerated development of atherosclerosis. Can. J. Diabetes.

[14] Zorio E., Navarro S., Medina P., Estelles A., Osa A., Rueda J., Cubillo P., Aznar J., Espana F. (2006). Circulating activated protein C is reduced in young survivors of myocardial infarction and inversely correlates with the severity of coronary lesions. J. Thromb. Haemost..

[15] Laszik Z.G., Zhou X.J., Ferrell G.L., Silva F.G., Esmon C.T. (2001). Down-regulation of endothelial expression of endothelial cell protein C receptor and thrombomodulin in coronary atherosclerosis. Am. J. Pathol..

[16] Isermann B., Vinnikov I.A., Madhusudhan T., Herzog S., Kashif M., Blautzik J., Corat M.A., Zeier M., Blessing E., Oh J. (2007). Activated protein C protects against diabetic nephropathy by inhibiting endothelial and podocyte apoptosis. Nat. Med..

[17] Matsumoto K., Yano Y., Gabazza E.C., Araki R., Bruno N.E., Suematsu M., Akatsuka H., Katsuki A., Taguchi O., Adachi Y. (2007). Inverse correlation between activated protein C generation and carotid atherosclerosis in Type 2 diabetic patients. Diabet. Med..

[18] Seehaus S., Shahzad K., Kashif M., Vinnikov I.A., Schiller M., Wang H., Madhusudhan T., Eckstein V., Bierhaus A., Bea F. (2009). Hypercoagulability inhibits monocyte transendothelial migration through protease-activated receptor-1-, phospholipase-Cbeta-, phosphoinositide 3-kinase-, and nitric oxide-dependent signaling in monocytes and promotes plaque stability. Circulation.

[19] Vukovich T.C., Schernthaner G. (1986). Decreased protein C levels in patients with insulin-dependent type I diabetes mellitus. Diabetes.

[20] Schneider J.G., Isermann B., Kleber M.E., Wang H., Boehm B.O., Grammer T.B., Prueller F., Nawroth P.P., Maerz W. (2014). Inverse association of the endogenous thrombin potential (ETP) with cardiovascular death: The Ludwigshafen Risk and Cardiovascular Health (LURIC) study. Int. J. Cardiol..

[21] Ardissino D., Merlini P.A., Bauer K.A., Galvani M., Ottani F., Franchi F., Bertocchi F., Rosenberg R.D., Mannucci P.M. (2003). Coagulation activation and long-term outcome in acute coronary syndromes. Blood.

[22] Borissoff J.I., Otten J.J., Heeneman S., Leenders P., van Oerle R., Soehnlein O., Loubele S.T., Hamulyak K., Hackeng T.M., Daemen M.J. (2013). Genetic and pharmacological modifications of thrombin formation in apolipoprotein e-deficient mice determine atherosclerosis severity and atherothrombosis onset in a neutrophil-dependent manner. PLoS ONE.

[23] Eitzman D.T., Westrick R.J., Shen Y., Bodary P.F., Gu S., Manning S.L., Dobies S.L., Ginsburg D. (2005). Homozygosity for factor V Leiden leads to enhanced thrombosis and atherosclerosis in mice. Circulation.

[24] Martini C.H., Doggen C.J., Cavallini C., Rosendaal F.R., Mannucci P.M. (2005). No effect of polymorphisms in prothrombotic genes on the risk of myocardial infarction in young adults without cardiovascular risk factors. J. Thromb. Haemost..

[25] Smiles A.M., Jenny N.S., Tang Z., Arnold A., Cushman M., Tracy R.P. (2002). No association of plasma prothrombin concentration or the G20210A mutation with incident cardiovascular disease: Results from the Cardiovascular Health Study. Thromb. Haemost..

[26] Borissoff J.I., Spronk H.M., Heeneman S., ten Cate H. (2009). Is thrombin a key player in the ‘coagulation-atherogenesis’ maze?. Cardiovasc. Res..

[27] Posthuma J.J., Posma J.J.N., van Oerle R., Leenders P., van Gorp R.H., Jaminon A.M.G., Mackman N., Heitmeier S., Schurgers L.J., Ten Cate H. (2019). Targeting Coagulation Factor Xa Promotes Regression of Advanced Atherosclerosis in Apolipoprotein-E Deficient Mice. Sci. Rep..

[28] Weiler-Guettler H., Christie P.D., Beeler D.L., Healy A.M., Hancock W.W., Rayburn H., Edelberg J.M., Rosenberg R.D. (1998). A targeted point mutation in thrombomodulin generates viable mice with a prethrombotic state. J. Clin. Investig..

[29] Shahzad K., Gadi I., Nazir S., Al-Dabet M.M., Kohli S., Bock F., Breitenstein L., Ranjan S., Fuchs T., Halloul Z. (2018). Activated protein C reverses epigenetically sustained p66(Shc) expression in plaque-associated macrophages in diabetes. Commun. Biol..

[30] Shahzad K., Bock F., Dong W., Wang H., Kopf S., Kohli S., Al-Dabet M.M., Ranjan S., Wolter J., Wacker C. (2015). Nlrp3-inflammasome activation in non-myeloid-derived cells aggravates diabetic nephropathy. Kidney Int..

[31] Madhusudhan T., Wang H., Dong W., Ghosh S., Bock F., Thangapandi V.R., Ranjan S., Wolter J., Kohli S., Shahzad K. (2015). Defective podocyte insulin signalling through p85-XBP1 promotes ATF6-dependent maladaptive ER-stress response in diabetic nephropathy. Nat. Commun..

[32] Bock F., Shahzad K., Wang H., Stoyanov S., Wolter J., Dong W., Pelicci P.G., Kashif M., Ranjan S., Schmidt S. (2013). Activated protein C ameliorates diabetic nephropathy by epigenetically inhibiting the redox enzyme p66Shc. Proc. Natl. Acad. Sci. USA.

[33] Madhusudhan T., Wang H., Ghosh S., Dong W., Kumar V., Al-Dabet M.M., Manoharan J., Nazir S., Elwakiel A., Bock F. (2017). Signal integration at the PI3K-p85-XBP1 hub endows coagulation protease activated protein C with insulin-like function. Blood.

[34] Gaul S., Shahzad K., Medert R., Gadi I., Mader C., Schumacher D., Wirth A., Ambreen S., Fatima S., Boeckel J.N. (2022). Novel Nongenetic Murine Model of Hyperglycemia and Hyperlipidemia-Associated Aggravated Atherosclerosis. Front. Cardiovasc. Med..

[35] Shahzad K., Thati M., Wang H., Kashif M., Wolter J., Ranjan S., He T., Zhou Q., Blessing E., Bierhaus A. (2011). Minocycline reduces plaque size in diet induced atherosclerosis via p27(Kip1). Atherosclerosis.

[36] Nazir S., Gadi I., Al-Dabet M.M., Elwakiel A., Kohli S., Ghosh S., Manoharan J., Ranjan S., Bock F., Braun-Dullaeus R.C. (2017). Cytoprotective activated protein C averts Nlrp3 inflammasome-induced ischemia-reperfusion injury via mTORC1 inhibition. Blood.

[37] Gadi I., Fatima S., Elwakiel A., Nazir S., Mohanad Al-Dabet M., Rana R., Bock F., Manoharan J., Gupta D., Biemann R. (2021). Different DOACs Control Inflammation in Cardiac Ischemia-Reperfusion Differently. Circ. Res..

[38] Clarke J.H., Light D.R., Blasko E., Parkinson J.F., Nagashima M., McLean K., Vilander L., Andrews W.H., Morser J., Glaser C.B. (1993). The short loop between epidermal growth factor-like domains 4 and 5 is critical for human thrombomodulin function. J. Biol. Chem..

[39] Carr M.E. (2001). Diabetes mellitus: A hypercoagulable state. J. Diabetes Complicat..

[40] Tripodi A., Branchi A., Chantarangkul V., Clerici M., Merati G., Artoni A., Mannucci P.M. (2011). Hypercoagulability in patients with type 2 diabetes mellitus detected by a thrombin generation assay. J. Thromb. Thrombolysis.

[41] Madhusudhan T., Ghosh S., Wang H., Dong W., Gupta D., Elwakiel A., Stoyanov S., Al-Dabet M.M., Krishnan S., Biemann R. (2020). Podocyte Integrin-beta 3 and Activated Protein C Coordinately Restrict RhoA Signaling and Ameliorate Diabetic Nephropathy. J. Am. Soc. Nephrol..

[42] Dong W., Wang H., Shahzad K., Bock F., Al-Dabet M.M., Ranjan S., Wolter J., Kohli S., Hoffmann J., Dhople V.M. (2015). Activated Protein C Ameliorates Renal Ischemia-Reperfusion Injury by Restricting Y-Box Binding Protein-1 Ubiquitination. J. Am. Soc. Nephrol..

[43] Neergaard-Petersen S., Hvas A.M., Kristensen S.D., Grove E.L., Larsen S.B., Phoenix F., Kurdee Z., Grant P.J., Ajjan R.A. (2014). The influence of type 2 diabetes on fibrin clot properties in patients with coronary artery disease. Thromb. Haemost..

[44] Kozakova M., Morizzo C., Goncalves I., Natali A., Nilsson J., Palombo C. (2019). Cardiovascular organ damage in type 2 diabetes mellitus: The role of lipids and inflammation. Cardiovasc. Diabetol..

[45] Randeria S.N., Thomson G.J.A., Nell T.A., Roberts T., Pretorius E. (2019). Inflammatory cytokines in type 2 diabetes mellitus as facilitators of hypercoagulation and abnormal clot formation. Cardiovasc. Diabetol..

[46] Ridker P.M., Libby P., MacFadyen J.G., Thuren T., Ballantyne C., Fonseca F., Koenig W., Shimokawa H., Everett B.M., Glynn R.J. (2018). Modulation of the interleukin-6 signalling pathway and incidence rates of atherosclerotic events and all-cause mortality: Analyses from the Canakinumab Anti-Inflammatory Thrombosis Outcomes Study (CANTOS). Eur. Heart J..

[47] Christoffersen M., Tybjaerg-Hansen A. (2021). Targeting IL-6 in patients at high cardiovascular risk. Lancet.

[48] Ridker P.M., Everett B.M., Thuren T., MacFadyen J.G., Chang W.H., Ballantyne C., Fonseca F., Nicolau J., Koenig W., Anker S.D. (2017). Antiinflammatory Therapy with Canakinumab for Atherosclerotic Disease. N. Engl. J. Med..

[49] Ridker P.M., Devalaraja M., Baeres F.M.M., Engelmann M.D.M., Hovingh G.K., Ivkovic M., Lo L., Kling D., Pergola P., Raj D. (2021). IL-6 inhibition with ziltivekimab in patients at high atherosclerotic risk (RESCUE): A double-blind, randomised, placebo-controlled, phase 2 trial. Lancet.

[50] Scheller J., Chalaris A., Schmidt-Arras D., Rose-John S. (2011). The pro- and anti-inflammatory properties of the cytokine interleukin-6. Biochim. Biophys. Acta.

[51] Gorbacheva L., Pinelis V., Ishiwata S., Strukova S., Reiser G. (2010). Activated protein C prevents glutamate- and thrombin-induced activation of nuclear factor-kappaB in cultured hippocampal neurons. Neuroscience.

[52] Joyce D.E., Gelbert L., Ciaccia A., DeHoff B., Grinnell B.W. (2001). Gene expression profile of antithrombotic protein c defines new mechanisms modulating inflammation and apoptosis. J. Biol. Chem..

[53] El-Osta A., Brasacchio D., Yao D., Pocai A., Jones P.L., Roeder R.G., Cooper M.E., Brownlee M. (2008). Transient high glucose causes persistent epigenetic changes and altered gene expression during subsequent normoglycemia. J. Exp. Med..

[54] Ott S.J., El Mokhtari N.E., Musfeldt M., Hellmig S., Freitag S., Rehman A., Kuhbacher T., Nikolaus S., Namsolleck P., Blaut M. (2006). Detection of diverse bacterial signatures in atherosclerotic lesions of patients with coronary heart disease. Circulation.

[55] Shen X., Li L., Sun Z., Zang G., Zhang L., Shao C., Wang Z. (2021). Gut Microbiota and Atherosclerosis-Focusing on the Plaque Stability. Front. Cardiovasc. Med..

[56] Bhakta-Guha D., Efferth T. (2015). Hormesis: Decoding Two Sides of the Same Coin. Pharmaceuticals.

[57] Isermann B. (2017). Homeostatic effects of coagulation protease-dependent signaling and protease activated receptors. J. Thromb. Haemost..

[58] Hayakawa M., Yamakawa K., Saito S., Uchino S., Kudo D., Iizuka Y., Sanui M., Takimoto K., Mayumi T., Ono K. (2016). Recombinant human soluble thrombomodulin and mortality in sepsis-induced disseminated intravascular coagulation. A multicentre retrospective study. Thromb. Haemost..

[59] Yamakawa K., Aihara M., Ogura H., Yuhara H., Hamasaki T., Shimazu T. (2015). Recombinant human soluble thrombomodulin in severe sepsis: A systematic review and meta-analysis. J. Thromb. Haemost..

[60] van Iersel T., Stroissnig H., Giesen P., Wemer J., Wilhelm-Ogunbiyi K. (2011). Phase I study of Solulin, a novel recombinant soluble human thrombomodulin analogue. Thromb. Haemost..

[61] Ryang Y.M., Dang J., Kipp M., Petersen K.U., Fahlenkamp A.V., Gempt J., Wesp D., Rossaint R., Beyer C., Coburn M. (2011). Solulin reduces infarct volume and regulates gene-expression in transient middle cerebral artery occlusion in rats. BMC Neurosci..

[62] Su E.J., Geyer M., Wahl M., Mann K., Ginsburg D., Brohmann H., Petersen K.U., Lawrence D.A. (2011). The thrombomodulin analog Solulin promotes reperfusion and reduces infarct volume in a thrombotic stroke model. J. Thromb. Haemost..

[63] Mosnier L.O., Gale A.J., Yegneswaran S., Griffin J.H. (2004). Activated protein C variants with normal cytoprotective but reduced anticoagulant activity. Blood.

[64] De Ceunynck K., Peters C.G., Jain A., Higgins S.J., Aisiku O., Fitch-Tewfik J.L., Chaudhry S.A., Dockendorff C., Parikh S.M., Ingber D.E. (2018). PAR1 agonists stimulate APC-like endothelial cytoprotection and confer resistance to thromboinflammatory injury. Proc. Natl. Acad. Sci. USA.

